# The risk factors for mental health disorders in patients with type 2 diabetes: An umbrella review of systematic reviews with and without meta-analysis

**DOI:** 10.1016/j.heliyon.2024.e28782

**Published:** 2024-04-05

**Authors:** Amani Busili, Kanta Kumar, Laura Kudrna, Idris Busaily

**Affiliations:** a College of Medical and Dental Sciences, University of Birmingham, Birmingham, United Kingdom; b Institute of Clinical Sciences, College of Medical and Dental Sciences, University of Birmingham, Birmingham, United Kingdom; c Institute of Applied Health Research, University of Birmingham, Birmingham, United Kingdom; d Lecture, College of Dentistry, Jazan University, Jazan, Kingdom of Saudi Arabia

**Keywords:** Mental health, Mental health disorders, Diabetes, Type 2 diabetes, T2DM, Risk factors, Umbrella review

## Abstract

**Background:**

Patients with type 2 diabetes have a nearly twofold higher rate of diagnosed mental disorders than those without diabetes. The association between type 2 diabetes and mental disorders is well established in the literature and recognized as a bidirectional relationship. This study aims to conduct an umbrella review of risk and protective factors for mental health disorders in patients with type 2 diabetes and assess the credibility of the evidence for the association between each factor and mental health disorders.

**Methods:**

A comprehensive search was conducted of Medline via PubMed, Web of Science, EMBASE, CINHAL, and PsycINFO from inception to November 17, 2022, to identify systematic reviews with and without meta-analyses examining associations of factors with mental health disorders in patients with type 2 diabetes. For each association, we recalculated the summary effect size and 95% confidence intervals using random-effects models. We also reported the 95% prediction interval and between-group heterogeneity.

**Results:**

The study included 11 systematic reviews that met the inclusion criteria, comprising eight meta-analyses and three without meta-analyses. This involved approximately 489,930 participants and encompassed 26 unique factors. Six factors were rated as having suggestive evidence at the Class III level. These factors were obesity (n = 18,456, OR 1.75 [1.2 to 2.59], I^2^ 97.7%), neuropathy (n = 3898, OR 2.01 [1.60 to 2.54], I^2^ 44.5%), diabetes complications (n = 1769, OR 1.90 [1.53 to 2.36], I^2^ 39.3%), peripheral blood concentrations of CRP (n = 1742, SMD 0.31 [0.16 to 0.45], I^2^ 84.1%), female sex (n = 35,162, OR 1.36 [1.19 to 1.54], I^2^ 64.5%), and social support (n = 3151, OR 2.02 [1.51 to 2.70], I^2^ 87.2%).

**Conclusions:**

Several factors associated with mental health disorders in patients with type 2 diabetes were identified with varying degrees of supporting evidence. Significantly, obesity, neuropathy, complications, peripheral blood CRP concentrations, female sex, and social support emerged with suggestive evidence. An investigation of these factors should be conducted to target interventions accordingly. It may be helpful to prioritize patients who have these risk factors as high-risk groups and to implement plans and policies to enhance support before mental health disorders occur.

## 1 Introduction

Type 2 diabetes is increasingly recognized as a multifaceted cardiorenal-metabolic condition characterized primarily by sustained high blood glucose levels due to chronic positive energy imbalances [1,2]. According to a recent study, it constitutes nearly 90% of the approximately 537 million cases of diabetes worldwide, with projections suggesting that this figure will increase to 783 million by 2045 [3]. A significant proportion of patients with type 2 diabetes reside in low- and middle-income countries [4], and the prevalence is increasing, particularly among children and young adults under the age of 40 [5].

However, type 2 diabetes increases the risk of both microvascular (e.g., retinopathy) and macrovascular (e.g., ischemic heart disease) complications. Factors such as prolonged diabetes duration, suboptimal glycaemic control, increased glycaemic variability, male sex, underlying comorbidities, and pre-existing complications such as albuminuria or subclinical atherosclerosis are all associated with an elevated risk of microvascular and macrovascular complications in patients with type 2 diabetes [[6], [7], [8]].

Moreover, in addition to its impact on physical health, type 2 diabetes also extends its influence on mental health. The association between type 2 diabetes and mental illness has been well established in the literature, and it is recognized as a bidirectional relationship [9,10]. For instance, patients with type 2 diabetes exhibit a nearly twofold higher rate of diagnosed mental illnesses compared to those without diabetes. For instance, Grigsby et al. (2002) found that approximately 40% of patients with type 2 diabetes exhibit heightened symptoms of anxiety [11]. The experience of living with diabetes often entails significant emotional stress, which can exert adverse effects on an individual's mental well-being. Moreover, the biological consequences of elevated blood sugar levels have been linked to the development of depression in individuals with diabetes. In the absence of adequate treatment, diabetes, poor mental health, and mental illnesses can negatively impact self-care practices and contribute to the deterioration of mental and physical health [12].

Conversely, both depression and schizophrenia are recognized risk factors for the onset of type 2 diabetes due to their impact on the body's resistance to insulin [13,14]. Individuals with mental illnesses often share several other risk factors for diabetes, including obesity and elevated cholesterol levels. Notably, antipsychotic medications have demonstrated a significant propensity to induce weight gain, with obesity rates reaching up to 3.5 times higher in individuals with serious mental illnesses than in the general population [15].

Despite the high treatability of mental disorders such as depression in diabetic patients, it remains unrecognized and untreated in approximately two-thirds of patients [16,17]. This is a concerning finding, especially given the higher suicide rates among patients with diabetes [18]. Patients with diabetes face twice the prevalence of depression throughout their lifespan compared to the general population [19]. A systematic review revealed that around one in four adults with diabetes experiences significant depressive symptoms [20], and 10%–15% of individuals with diabetes receive a formal diagnosis of depressive disorder [21].

Mental health disorders that co-occur with diabetes are associated with several negative consequences. These include decreased quality of life [22,23], increased healthcare costs [24], greater utilization of healthcare services [25], higher rates of hospitalization and absenteeism [26], and poorer treatment compliance [27]. Moreover, patients with co-occurring psychiatric and endocrine disorders have twice the level of healthcare costs compared to those without such disorders [28].

In terms of risk factors, research has highlighted the significant impact of biological factors on mental health in patients with type 2 diabetes. Genetic predisposition and obesity, in particular, have been shown to increase the risk of developing mental health disorders in this population [29,30]. Genetic factors play a role in susceptibility to both diabetes and mental health disorders, and individuals with a family history of these conditions may be at higher risk. Moreover, obesity, which is a common risk factor for type 2 diabetes, has been shown to contribute to the development of mental health disorders in patients with diabetes [30].

In addition to these factors, health-related behaviours also play a crucial role in the mental health of patients with type 2 diabetes. For example, physical inactivity has been linked to the development of mental health disorders in this population [31] and a lack of regular physical activity, often associated with sedentary lifestyles, can exacerbate both diabetes and mental health. Physical activity has been shown to have numerous mental health benefits, including reducing stress, improving mood, and enhancing overall well-being. Therefore, patients with type 2 diabetes who are physically inactive may be at higher risk for mental health disorders [32].

Various environmental factors may also contribute to the heightened risk of mental health disorders among patients with type 2 diabetes [33]. For instance, residing in neighbourhoods characterized by higher levels of deprivation has been found to be associated with an increased likelihood of experiencing depression and anxiety due to factors such as the stress of poverty and poor service accessibility [34]. Furthermore, the management of type 2 diabetes itself can impose significant psychological challenges. The daily demands of monitoring blood glucose levels, adhering to medication regimens, and managing lifestyle changes can lead to heightened stress and psychological distress [35]. Moreover, the fear of potential complications associated with diabetes, such as vision loss, kidney failure, and amputations, can exacerbate anxiety and impact mental well-being, as noted by Ref. [36].

Furthermore, social support and access to healthcare services also play a crucial role in the development and management of mental health disorders in patients with type 2 diabetes. Studies have shown that patients with diabetes who have limited social support are at increased risk of developing depression and anxiety [37]. Similarly, inadequate access to healthcare services can prevent patients with type 2 diabetes from receiving appropriate care for both their physical and mental health needs, which can exacerbate mental health disorders and lead to poorer overall health outcomes [19].

The biopsychosocial model, proposed by George and Engel in 1980, offers a comprehensive framework for understanding the complex interactions between biological, psychological, and social factors that contribute to the development and management of mental health disorders in patients with type 2 diabetes [38]. This model emphasizes that mental health disorders arise from a combination of biological, psychological, and social factors that interact with each other to affect an individual's mental health outcomes. In the context of type 2 diabetes, this means that genetic predisposition, obesity, physical inactivity, neighbourhood deprivation, daily demands of managing diabetes, and limited social support or healthcare access can all influence an individual's mental health outcomes.

However, some critics have argued that the biopsychosocial model may be too broad or too narrow in its application. Ghaemi (2009) suggested that the model can be overly eclectic, leading to a loss of scientific rigour and testability of hypotheses and allows practitioners to choose their preferred approach without considering all aspects of the framework [39]. In addition, Douzenis et al. (2009) argued that the model can be too narrowly applied, focusing on deficits and illness to the exclusion of individual values and autonomy [40]. Consequently, some researchers suggest incorporating a patient-centered approach into the biopsychosocial model. McKay et al. (2012) proposed that such an approach could avoid the pitfalls of a narrow application of the model and encourage clinicians to focus on patients' autonomy, values, and life goals [41]. They argued that this approach can promote recovery and a more holistic understanding of mental health. Despite these debates, supporters of the biopsychosocial model maintain that the model was always intended to be practiced within a value system that is empathic and respectful of autonomy [42].

With this background, the main aim of this umbrella review is to systematically investigate the factors associated with the development of mental health disorders in patients with type 2 diabetes. As indicated, various biological, psychological, and social factors can interact to contribute to the development and management of mental health disorders in this population.

Throughout this review, our preferred term is 'mental health disorders,' intentionally chosen over 'mental health illnesses or symptoms.' This encompasses both diagnosed conditions and associated symptoms, such as insomnia, recognizing that 'mental health disorders' extend beyond mere symptoms to represent specific diagnostic conditions. It is crucial to highlight that 'mental health illness' directly refers to a medical condition or disease. Our focus in this review is to identify factors associated with the most prevalent mental health disorders among patients with type 2 diabetes, including depression (specifically Major Depressive Disorder (MDD) and dysthymia). We emphasize studies that include patients with type 2 diabetes who subsequently develop mental health disorders after their diagnosis as shown in Table 1.

**Table 1:** PECO question.

PECOS Question	Inclusion Criteria	Exclusion Criteria
Population	Adults aged 18 years and older with type 2 diabetes (any sex or ethnicity).	Children, adolescents (<18 yars), pregnant women with gestational diabetes, and individuals with other chronic diseases
Exposure	Exposure to risk and protective factors such as genetic, socio-demographic, or environmental factors.	N/A
Comparison	When available, group those who have not been exposed to potential factors.	N/A
Outcome	Mental health disorders (depression, anxiety, insomnia, suicide, and suicidal ideation).	Articles with outcomes other than established mental health disorders, such as those related to severity of symptoms. Articles that examine diabetes (alone) as a risk factor for mental health disorders are excluded.
Study	Systematic reviews or meta-analyses of individual studies examining associations between factors and mental health disorders in patients with type 2 diabetes. Studies reporting enough data to perform the analyses. Efforts made to contact corresponding authors for missing data. Articles with no reply from corresponding authors are excluded.	Articles that investigate the association between mental health disorders and other types of diabetes, such as type 1 or gestational diabetes.
Language	English language	Another language
Setting	All settings	N/A
Date	From inception to Nov 17, 2022	N/A

Investigating factors associated with mental health disorders in patients with type 2 diabetes is clinically important for several reasons. First, these factors could potentially be used to improve the prediction of mental health disorders in those at risk of developing the disorder. Second, some of these factors may be modifiable through preventive interventions, which could lead to improved mental health outcomes. Finally, understanding these factors could inform outreach campaigns aimed at the patients with type 2 diabetes, promoting awareness of the risk and protective factors for mental health disorders and the importance of mental health care.

To understand why patients with type 2 diabetes are at risk for mental health disorders, it is necessary to examine the risk factors and underlying mechanisms that contribute to the development of these disorders. Moreover, a growing body of literature in the form of systematic reviews has suggested that mental health disorders in type 2 diabetes may stem from a range of different mechanisms [43]. However, there has been heterogeneity in the study designs among the primary studies, such as cross-sectional studies, and variation in the methodological quality of the systematic reviews, such as differences in inclusion criteria and data analysis approaches. Therefore, a standardized overview that includes a comprehensive quality assessment is needed.

It is important to note that the results of studies investigating factors associated with mental health disorders in individuals with type 2 diabetes may be influenced by various types of biases; for example, it is not possible to conduct randomized controlled trials to determine the causality of the identified factors, such as lifestyle factors and genetic predisposition, and most evidence is based on observational studies. Therefore, there is a need for rigorous and comprehensive quality assessment to reduce the impact of potential biases, such as selection bias and information bias, and increase the reliability of the findings. One way of achieving this is through the use of umbrella reviews which evaluate evidence from systematic reviews and meta-analyses, the method for which is now outlined.

## 2 Methods

The Preferred Reporting Items for Systematic Reviews and Meta-analyses (PRISMA) guidelines were followed when reporting this umbrella review [44]. The study has been registered in PROSPERO (CRD42023401416).

### 2.1 Literature search strategy

An umbrella review, in essence, is a systematic review of systematic reviews that systematically collects and evaluates existing summary information. To achieve optimal searching in the review [45], five electronic bibliographic databases were used: Medline via PubMed, Web of Science, EMBASE, CINHAL, and PsycINFO from inception to November 17, 2022 to identify systematic reviews with and without meta-analyses examining associations of factors with mental health disorders in patients with type 2 diabetes. There were no restrictions regarding the publication setting considered in this review. Backwards searching was conducted to identify reviews that may have been missed during the original electronic searches.

EndNote (https://www.myendnoteweb.com/) was used to store, help organize, and maintain all references and to ensure a systematic and comprehensive search. First, specific subject headings are identified in each database (such as MeSH terms, PsycINFO Thesaurus, and their synonyms). The Boolean operators "AND" and "OR" were used to combine search terms [46]. In the following step, the search strategy combined MeSH terms and keywords that were used in MEDLINE (via PubMed) and adjusted for the other electronic databases.

### 2.2 Inclusion and exclusion criteria

All systematic reviews with or without meta-analyses of quantitative individual studies, including observational studies (e.g., case‒control, cohort, and cross-sectional studies) and randomized controlled trials (RCTs), were included. The studies examined associations between factors, such as genetic, socio-demographic, or environmental factors, and mental health outcomes (depression, anxiety, insomnia, suicide, and suicidal ideation) in patients with type 2 diabetes. Studies included a comparison group that represented non-exposure to the factors being examined, and they reported enough data to perform the analyses. In cases where data were not available, the corresponding author was contacted for additional information. If no reply was received, the article was excluded.

If two articles presented overlapping datasets and examined the same factor, only the article with the largest dataset was retained for the main analysis based on criteria such as sample size and study quality.

### 2.3 Study selection

A summary of the Population (P), Exposure (E), Comparators (C) and Outcomes (O) considered, following the PECO acronym, is shown in Table 1.

Participant/Population Characteristics: The umbrella review included adults aged 18 years and older who were diagnosed with type 2 diabetes, without any restrictions based on gender, ethnicity, or setting. The diagnosis of T2DM was confirmed through self-report by physicians, medical records, or glucose testing, following the criteria of fasting plasma glucose ≥7.0 mmol/L and/or 2-h postprandial plasma glucose ≥11.1 mmol/L as defined by the World Health Organization [47]. For studies that did not specify the type of diabetes, attempts were made to contact the original authors to obtain information on diabetes type, age group, and outcomes of interest. In cases where no response was received, the article was excluded from the review.

Study Design: Systematic reviews with or without meta-analyses of quantitative individual studies, including observational studies (e.g., case‒control, cohort, and cross-sectional), and randomized controlled trials (RCTs) were included.

Types of Outcomes: Articles that investigate the association between risk factors such as genetic, socio-demographic, or environmental factors and mental health outcomes, including depression, anxiety, insomnia, suicide, and suicide ideation. Mental health disorders at baseline were determined by 1) a diagnostic interview, 2) data from medical record documentation or registries, 3) medication prescription as a proxy measurement, 4) self-reported psychiatric disorders, or 5) elevated levels of clusters of psychiatric symptoms (e.g., questionnaires).

Exclusion criteria: These were established to maintain the rigour and relevance of the research findings. Studies that met any of the following conditions were excluded from the analysis: 1) studies that investigated the association between mental health disorders and other types of diabetes, such as type 1 or gestational diabetes; 2) studies that examine type 2 diabetes as a risk factor for mental health disorders 3) studies that were not in English; and 4) studies conducted with children, adolescents, and pregnant women with gestational diabetes.

### 2.4 Data screening and extraction

All publications identified in the research for inclusion in the review were screened. The titles and abstracts of the search results were then screened according to the inclusion criteria, and the publications were coded as 'retrieve' or 'do not retrieve'. Subsequently, the retrieved studies were reviewed in full text to identify studies for inclusion. The number of records identified, duplicates removed, records screened and included or excluded, full-text articles assessed and included or excluded, and finally studies included in the narrative synthesis were reported using a PRISMA flow diagram.

For each eligible systematic review, the following data were extracted: (i) first author's name; (ii) year of publication; (iii) the examined risk factors; (iv) time sequence/direct link; and (v) number of included studies. If a quantitative synthesis of the evidence was performed, the summary effect size (ES) estimate (risk ratio (RR), odds ratio (OR), hazard ratio (HR) or incident risk ratio, with 95% confidence intervals (CIs)) was recorded. Data screening and extraction were conducted independently by two reviewers (AB, IB), who cross-checked each other's data.

### 2.5 Risk of bias assessment

The risk of bias assessment in this umbrella review utilized the Joanna Briggs Institute (JBI) checklist for the critical appraisal of systematic reviews and research syntheses [48]. This checklist consists of ten items that evaluate various methodological aspects of a systematically conducted review, including the clarity and explicitness of the review question, appropriateness of search strategies, approach to synthesizing evidence, potential sources of bias, and prospects for future research and policy making. Each item is assessed as "yes," "no," "unclear," or "not applicable." For each "yes" answer, one point is assigned. Studies included in this review were categorized as low, medium, or high-quality based on the total points obtained on the checklist. Studies scoring zero to four points were categorized as low-quality, five to seven points as medium-quality, and eight to ten points as high-quality. Risk of bias assessment was conducted independently by two reviewers (AB and IB), and any conflicts were addressed through discussion between them.

### 2.6 Statistical analysis

The statistical analysis in this review involved recalculating pooled effect sizes and their 95% confidence intervals (CIs) using random-effects models for each identified potential factor associated with mental health disorders based on available effect sizes from individual primary studies [49]. Between-heterogeneity was assessed using the I^2^ statistic, with a value of I^2^ > 50% indicating large heterogeneity [50,51]. Additionally, 95% prediction intervals for the summary effect sizes were reported to reveal the uncertainty of the effect sizes of the identified association [50].

Considering potential publication bias, small-study effects were assessed. Egger's regression asymmetry test was used to evaluate the presence of small-study effects [52], with a p value of less than 0.10 indicating that small studies may exaggerate the summary effect sizes compared to larger studies.

The umbrella review, which included eight systematic reviews, did not utilize meta-regression in order to examine potential sources of heterogeneity. This decision was made as a result of the nature of our research question as well as the availability of data. We examined potential factors associated with mental health disorders across a wide range of systematic reviews, which resulted in a considerable diversity of populations, interventions, and study designs. As a result of the high degree of heterogeneity and the relatively small number of reviews included, it was not possible to conduct meaningful meta-regression with limited data. In order to address potential sources of variability, we relied on other standard approaches, such as heterogeneity assessment using the I^2^ statistic. Due to the limited availability of eligible systematic reviews that met our predefined inclusion criteria, we did not conduct sensitivity analyses. The relatively small number of reviews in our study made it impractical to perform extensive sensitivity assessments. Nevertheless, we aimed to provide a comprehensive overview of the existing evidence, acknowledging the limitations associated with the available data.

A classification system was used to assess the credibility of evidence based on a previously published umbrella review [53,54]. The levels of evidence were categorized into different classes, including convincing (Class I), highly suggestive (Class II), suggestive (Class III), weak (Class IV), or non-significant (NS). The criteria for each class were as follows: (i) Class I: studies with over 1000 cases, significant summary associations (P < 10^6) per random-effects calculation, no evidence of small-study effects, no evidence of excess of significance bias, prediction intervals not including the null, and low heterogeneity (I^2^ < 50%); (ii) Class II: studies with significant summary associations (P < 10^6) per random-effects calculation, over 1000 cases, and the largest study with a 95% confidence interval excluding the null; (iii) Class III: studies with over 1000 cases and significant summary associations (P < 10^3) per random-effects calculation; (iv) Class IV: all other risk factors with P < 0.05; and (v) non-significant associations: all associations with P > 0.05.

## 3 Results

**Fig. 1:**
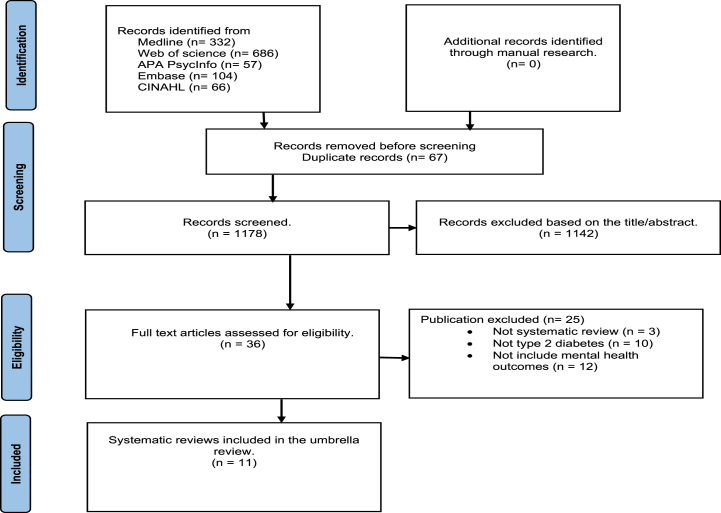
PRISMA-guided search strategy.

In total, 1178 records were screened. After removing duplicate records (n = 67), 1142 records were excluded based on title and abstract screening. Full text assessment was conducted on 36 articles for eligibility, out of which 25 publications were excluded for various reasons, including ineligible study designs (n = 3), ineligible outcomes (n = 12), and ineligible populations (n = 10). In total, 11 systematic reviews met the inclusion criteria and were included in the review. Among these, eight studies included meta-analyses, while three others did not employ meta-analysis in their approach (Fig. 1).

**Fig. 2:**
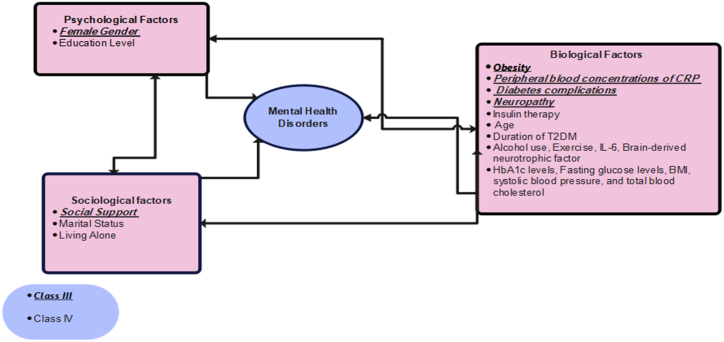
Mental health risk factors in type 2 diabetes: A biopsychosocial model.

Table 2 summarizes the characteristics of the included reviews with meta-analyses, comprising eight studies published in the last 10 years [[55], [56], [57], [58], [59], [60], [61], [62]]. These studies focused on mental health outcomes in patients with type 2 diabetes, specifically depression in seven studies and insomnia in one study. A total of 26 factors were identified as associated with mental health disorders in these patients, as shown in Fig. 2. The quality assessment using the JBI checklist rated two studies as medium quality, five studies as high quality, and one study as low quality.

**Table 2:** Characteristic of systematic reviews with meta-analysis included in the umbrella review.

Study	Date	Mental Health Outcome and (*Assessment Method*)	Factor	Time Sequence/Direct Link	Number of Primary Studies	JBI Quality
Bai (https://doi.org/10.1136/bmjopen-2017-020062)	2018	Depression *(PHQ, BDI, CES-D, DSM/ICD)*	Insulin Therapy	Type 2 Diabetes→Depression	12	High
Azmiardi 10.3961/jpmph.21.490	2022	Depression *(NA)*	Social Support	Type 2 Diabetes→Depression	11	High
Bartoli (https://doi.org/10.1002/gps.4397)	2016	Depression *(Depression symptoms scale with a cutoff value for clinical depression)*	Neuropathy	Type 2 Diabetes→Depression	13	Medium
Liu (https://doi.org/10.3389/fmed.2022.759499)	2022	Depression (*Depression scales, major depressive* disorder diagnosed *by operationalized criteria such as the Diagnostic and Statistical Manual of Mental Disorders (DSM) or the International Statistical Classification of Diseases and Related Health Problems* 10th *Revision (ICD-10).*	(Gender, Age, Education Level, Duration of T2DM, Complication, Smoking, Marital Status, Living Status, Alcohol Use)	Type 2 Diabetes→Depression	(18, 7, 8, 5, 6, 8, 7, 4, 6)	High
Simayi 10.1507/endocrj.EJ18-0579	2019		Exercise	Type 2 Diabetes→Depression	5	Low
Nguyen (https://doi-org.bham-ezproxy.idm.oclc.org/10.1016/j.psyneuen.2021.105448)	2021	Depression *(Validated screening tools for depression with appropriate cut-offs for scores)*.	Inflammatory Biomarker (Peripheral Blood Concentrations of CRP, IL-6, Brain Derived Neurotrophic Factor)	Type 2 Diabetes→Depression	(17,5,3)	High
Koopman 10.1210/clinem/dgz065	2019	Insomnia or Insomnia symptoms *(The PSQI questionnaire with a score of >5 points, self-report on having insomnia, the Insomnia Severity Index and the Medical Outcomes Study - Sleep Scale).*	Metabolic Parameters and Glycemic Control (HbA1c, FBG, BMI, Triglyceride, HDL, LDL, SBP, DSP, Total Blood Cholesterol)	Type 2 Diabetes→Insomnia (Symptoms)	(7, 11, 14, 8, 7, 7, 9, 8, 5)	High

Note: T2DM: Type 2 Diabetes Mellitus, CRP: C-reactive Protein, IL-6: Interleukin-6, HbA1c: Haemoglobin A1c, FBG: Fasting Blood Glucose, BMI: Body Mass Index, LDL: Low-Density Lipoprotein, HDL: High-Density Lipoprotein, SBP: Systolic Blood Pressure, DSP: Diastolic Blood Pressure, JBI: Joanna Briggs Institute checklist for the critical appraisal of systematic reviews and research syntheses, PHQ: Patient Health Questionnaire, BDI: Beck Depression Inventory, CES-D: Centre for Epidemiologic Studies–Depression Scale, DSM/ICD: Diagnostic and Statistical Manual of Mental Disorders/International Classification of Diseases, PSQI: Pittsburgh Sleep Quality Index.

Table 3 provides a concise summary of three systematic reviews that investigated the factors associated with depression in individuals with type 2 diabetes mellitus. These reviews did not conduct meta-analyses and had different study designs, including cross-sectional studies, intervention studies, and a combination of cross-sectional and trial studies [[63], [64], [65]]. The authors of these reviews concluded that depression in type 2 diabetes is linked with various biopsychosocial and nutritional factors, as well as physical and outdoor activities. Specifically, physical inactivity was found to increase the risk of depression based on findings from both cross-sectional and trial studies. The quality assessment of these systematic reviews varied from medium to high, indicating the reliability and validity of the evidence presented.

**Table 3:** Characteristics and evidence from systematic reviews without meta-analysis of the studies included.

First Author	Year	Title	Study Design	Outcome	N of Studies	Sample Size	Main Findings	JBI assessment
Amsah,10.3390/ijerph19084888	2022	Biopsychosocial and Nutritional Factors of Depression among Type 2 Diabetes Mellitus Patients: A Systematic Review	Cross-sectional	Biopsychosocial and nutritional factors associated with depression	19	8161	Biological (ge, female gender, long duration of diabetes, body mass index, HBA1c, presence of comorbidities, diabetic complication, and sexual dysfunction, psychological (Fear of diabetic complications, anxiety, mild impairment cognitive, low satisfaction with current treatment), social (physical inactivity, consumption of alcohol, poor social support, low educational status, smoking habit, marital status, single/un partnered ethnicity (Chines), living arrangement low socioeconomic status), and nutritional factors (lower albumin level (poor nutritional status) were shown to be linked with depression among T2DM patients.	Medium
Lysy https://doi.org/10.1111/j.1464-5491.2008.02545.x	2008	The association of physical activity and depression in type 2 diabetes	Cross-sectional, cohort and trials	Physical activity and depressed mood in Type 2 diabetes	12	–	In adults with Type 2 diabetes, the inactive are 1.72–1.75 times more likely to be depressed than the more active; the depressed are 1.22–1.9 times more likely to be physically inactive than the non-depressed. Two randomized trials demonstrated that a depression management program improved mood, but only one demonstrated increased physical activity.	Medium
Fraser 10.1093/heapro/daz064	2020	Psychological effects of outdoor activity in type 2 diabetes: A review	Intervention	Investigated whether conducting physical activity in outdoor environments had any effect on psychological problems (depression, anxiety, QOL) in individuals with type 2 diabetes	4	231	No significant effect was found for depression. However, general wellbeing was improved. Due to the limited number of studies eligible for inclusion and the heterogeneity of outcome measures, it was difficult to draw firm conclusions.	High

The grading and results of meta-analyses, as presented in Table 4, reveal that the sample sizes ranged from 1287 to 331,085. Most of the included meta-analyses reported statistically significant associations between the investigated factors and mental health outcomes, specifically depression and insomnia, in patients with type 2 diabetes.

**Table 4:** Risk and protective factors for mental health in patient with type 2 diabetes.

Factors	Measure metric	Random effects summary estimate (95% CI)	Random effects P value	N of Cases (Sample size)	I^2^ (%)	95% Prediction interval (PI)	Egger test P value	Small-study effects or excess significance bias	Class of evidence
Insulin therapy	OR	1.41 (1.13–1.76)	0.003	NR (331085)	69.7%	0.68 to 2.88	0.94	Neither	IV
Obesity	OR	1.75 (1.19–2.59)	0.001	18,456 (48,466)	97.7%	0.24 to 12.84	0.03	SSE and ESB	III
Social support	OR	2.02 (1.51–2.70)	0.0001	NR (3151)	87.2%	0.78 to 5.21	0.04	SSE and ESB	III
Neuropathy	OR	2.01 (1.60–2.54)	0.0001	(NR)					
3898	44.5%	1.08 to 3.78	0.06	Neither	III				
Gender (female vs male)	OR	1.36 (1.19–1.54)	0.0001	35162 (60100)	64.5%	0.88 to 2.09	0.10	ESB	III
Age (as risk factor for depression) (≥60 years old vs. <60 years old	OR	1.56 (1.13–2.13)	0.006	3210 (5925)	78.8%	0.59 to 4.11	0.88	ESB	IV
Education Level (Primary school or lower education level vs. College degree or higher education level)	OR	1.84 (1.16–2.92)	0.004	2259 (3131)	71.5%	0.46 to 7.30	0.81	ESB	IV
Duration of T2DM ((≥10 years vs. <5 years	OR	1.68(1.11–2.54)	0.01	565 (1287)	54.9%	0.46 to 6.14	0.97	ESB	IV
Complication	OR	1.90 (1.53–2.36)	0.000	1769 (3821)	39.3%	1.11 to 3.25	0.02	SSE	III
Smoking (current smoker vs nonsmoker	OR	0.85 (0.60–1.20)	0.36	1261 (7323)	78.4%	0.28 to 2.62	0.10	ESB	NS
Marital status (Abnormal marital status vs. normal marital status)	OR	1.39 (0.90–2.14)	0.14	960 (6238)	83.6%	0.33 to 5.83	0.96	ESB	NS
Living Status (living alone vs not living alone)	OR	2.76 (1.71–2.98)	0.0001	257 (1761)	0.0%	NR	0.73	Neither	IV
Alcohol using (Current alcohol user vs. non-drinker)	OR	0.70 (0.58–0.86)	0.001	954 (6717)	16.2%	0.48 to 1.04	0.34	Neither	IV
Exercise (regular vs irregular)	OR	0.51 (0.27–0.96)	0.04	1404 (3262)	91.8%	0.04 to 5.75	0.27	ESB	IV
Peripheral blood concentrations of CRP	SMD	0.31(0.16–0.45)	0.0001	1742 (16986)	84.1%	−0.28 to 0.89	0.677	ESB	III
IL-6	SMD	0.17 (0.04–0.30)	0.01	677 (5026)	48.4%	−0.21 to 0.55	0.08	Neither	IV
Brain derived neurotrophic factor	SMD	−0.37 (−0.64 to −0.10)	0.008	358 (1870)	61.5%	−3.27 to 2.54	0.967	ESB	IV
HbA1c levels	RR	1.18 (1.0–1.4)	0.02	NR	73.5%	0.77 to 1.81	0.146	ESB	IV
Fasting glucose levels	MD	0.40 (0.2–0.7)	0.002	NR	56.5%	0.32 to 1.11	0.428	ESB	IV
BMI	MD	0.38 (0.1–0.7)	0.02	NR	47.3%	0.51 to 1.28	0.842	Neither	IV
Triglyceride	MD	0.16 (−0.13, 0.45)	0.27	NR	91.3%	−0.82 to 1.14	NA	ESB	NS
HDL	MD	0.02 (−0.01, 0.05)	0.22	NR	0.0%	NA	NA	Neither	NS
LDL	M7D	0.05 (−0.07, 0.17)	0.43	NR	48.6%	−0.25 to 0.35	NA	Neither	NS
Systolic blood pressure	MD	2.69 (0.1–5.3)	0.05	NR	60.2%	5.23 to 10.62	0.683	Neither	IV
Diastolic blood pressure	MD	1.13 (−0.09, 2.34)	0.07	NR	25.5%	−1.48 to 3.74	NA	Neither	NS
Total blood cholesterol	MD	0.15 (0.03–0.3)	0.01	NR	0.0%	NA	0.891	Neither	IV

CI: Confidence Interval; OR: Odds Ratio; RR: Relative Risk; SMD: Standardized Mean Difference; MD: Mean Difference; ESB: Excess Significance Bias; SSE: Small-Study Effects; NA: Not Available; NR: Not Reported; N: Number of Cases (sample size); I2: I-squared statistic (a measure of heterogeneity); PI: Prediction Interval; CRP: C-reactive Protein; IL-6: Interleukin-6; HbA1c: Glycated Haemoglobin; BMI: Body Mass Index; HDL: High-Density Lipoprotein; LDL: Low-Density Lipoprotein; III: Class of evidence is rated as "III" (suggestive); V: Class of evidence is rated as "IV" (low quality evidence); NS: Class of evidence is rated as "NS" (not significant).

## 4 Risk factors for depression in patients with type 2 diabetes

### 4.1 Physical-related factors

Among the twelve physical-related factors evaluated, eleven demonstrated statistically significant summary effects (P < 0.05). These significant factors include higher risk among those with obesity (n = 18,456, OR 1.75 (1.19–2.59), I^2^ 97.7%), neuropathy (n = 3898, OR 2.01 (1.60–2.54), I^2^ 44.5%), diabetes complications (n = 1769, OR 1.90 (1.53–2.36), I^2^ 39.3%), and peripheral blood concentrations of CRP (n = 1742, SMD 0.31 (0.16–0.45), I^2^ 84.1%), all of which provide suggestive evidence of higher risk at the class III level.

Other factors, including insulin therapy, age, duration of T2DM, alcohol use, exercise, IL-6, and brain-derived neurotrophic factor, were rated as weak Class IV evidence. However, for the remaining associations, no statistically significant evidence was found (Table 4).

### 4.2 Psychological-related factors

Two psychological-related factors were examined. The association with female gender was rated as Class III (n = 35162, OR 1.36 (1.19–1.54), I^2^ 64.5%), while education level (primary school or lower education level vs. college degree or higher education level) showed weak evidence (Class IV) as a risk factor for depression (Table 4).

### 4.3 Social-related factors

For socio-demographic factors, three associations were examined. Social support (n = 3151, OR 2.02 (1.51–2.70), I^2^ 87.2%) was rated as suggestive (Class III). No association showed convincing (Class I) or highly suggestive (Class II) evidence. Marital status and living alone were evaluated as weak (Class IV) (Table 4).

## 5 Risk factors for insomnia in patients with type 2 diabetes

### 5.1 Physical-related factors

Only physically related factors were evaluated as risk factors for insomnia in patients with type 2 diabetes. Nine factors were examined, and no association was rated from Class I to Class III. HbA1c levels, fasting glucose levels, BMI, systolic blood pressure, and total blood cholesterol showed weak evidence (Class IV) as risk factors for insomnia. The remaining factors showed no significant association (Table 4).

## 6 Discussion

The research comprised 11 systematic reviews encompassing 26 outcomes, involving a comprehensive analysis of 273 studies with a combined participation of 489,930 individuals. The results of the umbrella review indicate that several risk factors show statistical significance in association with mental health disorders among patients with type 2 diabetes. Significant factors, however, contributing to a higher risk of developing mental health disorders, specifically depression, include obesity, neuropathy, female sex, the presence of diabetes complications, and elevated levels of inflammatory biomarkers such as C-reactive protein (CRP), as compared to other relevant factors. On the other hand, increased levels of social support and regular exercise are identified as the most important protective factors associated with a reduced risk of depression in patients with type 2 diabetes.

However, the majority of the evidence in the literature is derived from cross-sectional studies, which focus on the association between different factors and depression in patients with type 2 diabetes. Additionally, several conditions could potentially act as either risk factors or protective factors for mental health disorders in these patients.

Our findings concerning the factors contributing to mental health disorders in patients with type 2 diabetes carry substantial significance for several reasons. First, the prevalence of depression and insomnia in adults and older individuals with type 2 diabetes is a significant concern. Studies have estimated that depression, which is often associated with negative health outcomes, is highly prevalent globally, for example, in China (23.36%) [66]. Furthermore, research has documented that individuals with type 2 diabetes often experience poor sleep quality and sleep disorders, specifically insomnia, which can have a negative impact on their quality of life, diabetes self-care behaviours, and patient-reported outcomes [67].

The umbrella review confirmed that the prevalence of mental health disorders in patients with type 2 diabetes is associated with several risk factors. These factors should be considered if screening for mental health in this population and, where possible, prevented or modified. One of the key risk factors is metabolic disorders, including obesity, although this is a challenging risk factor to modify. Our review highlights the potential impact of these metabolic disorders on mental health outcomes in patients with type 2 diabetes. Several studies have reported similar associations between obesity and depression in patients with type 2 diabetes. Our reanalysis of existing data adds to the body of evidence, revealing suggestive evidence (class III) with low bias supporting the association between obesity and depression in this population.

This suggests that type 2 diabetes mellitus, obesity, and depression are clinical conditions that may share common anatomical substrates and physiological processes [68]. The association between type 2 diabetes, depression, and obesity is believed to be due to overlapping biological links, such as elevated cytokine levels, impaired neurotransmitter metabolism due to insulin deficiency, and hyperactivity of the hypothalamus-pituitary-adrenal (HPA) [69]. However, chronic stress can also lead to gut microbiota inflammation and hormonal imbalances, increasing the risk of developing these conditions [70].

Hence, based on this evidence, healthcare providers should prioritize patients with type 2 diabetes and obesity as those at risk of developing depression. Future interventions may necessitate innovative approaches, such as personalized lifestyle plans, psychological interventions, and health-promoting environments, to address this intricate interplay. Furthermore, research indicates that the stigma associated with obesity can also lead to adverse mental health outcomes among patients with type 2 diabetes [71]. Therefore, weight-reduction programmes for these patients may yield positive effects on their mental health [72]. Healthcare providers may need to broaden their focus beyond solely considering obesity as a risk factor for depression in patients with type 2 diabetes. Instead, they should expand their investigations to prioritize factors contributing to obesity, including certain economic and socio-demographic variables such as income and gender [73,74]. By adopting this approach, healthcare providers aim not only to address depression in patients with obesity but also to address the root causes of obesity in patients with type 2 diabetes.

Furthermore, this review confirmed that there is suggestive evidence, with low bias, supporting the association between neuropathy and depression in patients with type 2 diabetes. The literature extensively documents the association between neuropathy and depression in patients with type 2 diabetes [75]. In their study, Alghafri, Gatt, and Formosa (2020) found that patients with diabetic peripheral neuropathy had a higher prevalence of depression symptoms than those without neuropathy [76]. Neuropathy is a condition characterized by peripheral nerve damage, resulting in sensory disturbances such as numbness, tingling, and pain in the extremities. It is a common complication of diabetes, with at least 50% of individuals with diabetes developing diabetic neuropathy over time [77]. As a result, individuals with diabetic neuropathy may be at heightened risk of developing comorbid depression. Consequently, it is crucial to protect patients with diabetes from developing neuropathy and include depression screening, support and treatment when addressing neuropathy in these patients.

The results of this review indicate a suggestive level of evidence (class III) supporting a connection between depression and complications in patients with type 2 diabetes, which is in line with the findings of a previous systematic review and meta-analysis of longitudinal studies. An earlier review revealed a bidirectional relationship between depression and diabetes complications, with depression linked to an increased risk of macrovascular and microvascular complications and diabetes complications associated with a higher risk of depression [78]. Recent research has identified biological processes, including obesity, insulin resistance, and poor glucose control, as potential contributors to the development of diabetes complications in the presence of depression. Additionally, inflammatory processes have been proposed as a possible mechanism, as they have been linked to both diabetes and depression [79,80]. Therefore, healthcare providers may need to prioritize the mental well-being of patients with type 2 diabetes who are also experiencing complications. This can be achieved through the implementation of behavioural interventions and psycho-education programmes that specifically target coping strategies, stress management, and emotional well-being.

Furthermore, our research highlights the significance of nonmedical factors in relation to depression in patients with type 2 diabetes. These factors include female gender, social support, demographic and socioeconomic status, and individual behaviours such as alcohol use and physical activity levels. Our findings suggest that these factors may play a role in the development and severity of depression in patients with type 2 diabetes.

Socioeconomic factors, including living status, are pertinent to our findings. Our results indicate that patients with type 2 diabetes who live alone may exhibit a higher prevalence of depression, as evidenced by the grading system (class IV). Despite being classified as IV, we cannot overlook its significance. While this factor may not directly correlate with mental health disorders compared to others such as social support, it could indirectly influence factors contributing to mental health disorders in patients with type 2 diabetes.

In addition, our findings suggest that people with type 2 diabetes who have low social support may have a higher prevalence of depression, as indicated by suggestive evidence (class III) based on the grading system. Social support improves mental health and quality of life by helping people feel valued and connected to their social networks [81]. This sense of belonging is connected to fewer mental health difficulties and thus serves as a strategy to prevent depression. It is important for healthcare professionals to screen the level of social support among patients with type 2 diabetes as a first step to identify patients who are at risk of developing mental health disorders, such as older patients who have decreased social connections [82]. Establishing community support networks and peer groups for these patients can help mitigate feelings of isolation and offer valuable emotional support. Healthcare providers can play a proactive role in fostering these connections by either organizing support groups themselves or referring patients to available community resources.

Our reanalysis of existing data adds to the body of evidence, revealing suggestive evidence (Class III) with low bias that supports the association between female gender and depression in the context of type 2 diabetes. The review suggests that females with type 2 diabetes may have a higher prevalence of depression than males with type 2 diabetes. This may be attributed to differences in health behaviours, such as coping mechanisms, social support, and help-seeking behaviours, which can impact the risk of depression in females with type 2 diabetes. Additionally, women may face unique psychosocial challenges such as caregiving responsibilities, societal expectations, and discrimination, which may contribute to the higher prevalence of depression in females with type 2 diabetes [83]. According to Salk, Hyde, and Abramson (2017), the gender difference in depression declined in early adulthood and then remained relatively stable, remaining between OR = 1.71–2.02 [84]. Furthermore, this could be explained in part by the fact that women experience significant hormonal changes during pregnancy and postpartum; during the perinatal period, women with diabetes had two times more depression than their non-diabetic peers [85]. However, it is important to acknowledge that the relationship between sex, type 2 diabetes, and depression is complex and likely multifactorial [86,87]. Despite this complexity, there is a growing emphasis on empowering women with type 2 diabetes to prioritize and address their mental well-being.

Additionally, our reanalysis of available data contributes to the existing body of evidence, revealing suggestive evidence with low bias that support the association between inflammatory biomarkers, such as C-reactive protein (CRP), and depression in patients with type 2 diabetes (class III). However, it is important to note that this evidence is limited due to the small number of studies used to support this finding.

This connection between depression, inflammatory biomarkers, and type 2 diabetes may be bidirectional, as depression could trigger physiological changes that increase inflammation, and increased inflammation could also exacerbate depressive symptoms. Therefore, inflammatory biomarkers may play an important role in both the development and detection of depression in patients with type 2 diabetes. An examination of the mental health status of patients with diabetes who also suffer from inflammatory diseases may be warranted considering this association.

In addition, the findings of this review substantiate the link between metabolic parameters, specifically fasting blood glucose, HbA1c, BMI, and systolic blood pressure, and the increased risk of insomnia in patients with type 2 diabetes. However, it should be emphasized that the quality of evidence supporting this association was rated as weak according to the grading system employed. Interestingly, the consistency of these findings with other literature is mixed in regard to the relationship between sleep duration and HbA1c levels in patients with type 2 diabetes. Some studies suggest that short sleep duration (≤6 h/day) and frequent insomnia complaints may not be strongly associated, or may not be associated at all, with HbA1c levels in this population [88].

Furthermore, the lack of association between triglycerides, LDL (low-density lipoprotein), and HDL (high-density lipoprotein) levels, as potential symptoms of metabolic syndrome, and insomnia is a noteworthy finding in this review. The possible reason for this lack of association could be attributed to the methodology employed in the meta-analysis, which utilized a cross-sectional design and included a limited number of studies. The inclusion of a small number of studies in the meta-analysis may have further implications on the findings. A limited sample size may result in reduced statistical power, which can affect the ability to detect significant associations. It is possible that the meta-analysis may not have captured the full range of available evidence on the topic, which could impact the validity and generalizability of the findings. Hence, the findings of this study should be interpreted with caution and considered uncertain, with the possibility of changes in future research.

The current study's finding of no association between insomnia and triglycerides, LDL, and HDL levels is consistent with other studies in the literature. For instance, a population-based cross-sectional study utilizing data from the 2005–2008 United States National Health and Nutrition Examination Surveys found no significant relationship between insomnia symptoms and dyslipidemia [89].

Finally, the evidence from systematic reviews without meta-analysis suggests that several factors have been identified as potential contributors to depression in individuals with type 2 diabetes mellitus, including biological, psychological, social, and nutritional factors. These findings are consistent with previous literature that has highlighted the multifactorial nature of depression in patients with diabetes. The results from the systematic review also support the significant association between physical inactivity and depression in individuals with Type 2 diabetes, with inactive individuals being 1.72 to 1.75 times more likely to be depressed than those who are more physically active. Additionally, the review suggests that while there may not have been a significant effect on depression, there was an improvement in general well-being with the use of green exercise in individuals with Type 2 diabetes. This is in line with previous literature that has reported potential benefits of nature-based or green exercise on mental health outcomes. However, further research is needed to establish the effects of green exercise on depression in individuals with type 2 diabetes. The systematic review provides valuable insights and sets the stage for future studies to explore the role of green exercise as a potential adjunctive intervention for improving mental health outcomes in this population.

## 7 Limitations

Several limitations must be considered when interpreting the results of this review. First, our primary objective was to examine the risk factors associated with mental health disorders in patients with type 2 diabetes. There is, however, a notable limitation in this regard. While searching for relevant research, it became apparent that most of the included studies were primarily focused on depression, and there were no studies that met the inclusion criteria for anxiety, suicide, or suicidal ideation. This limitation emphasizes the need for further comprehensive investigations into the less explored areas of mental health disorders in individuals with type 2 diabetes, such as anxiety and suicide-related outcomes, to gain a deeper understanding of the complex interrelationship between these conditions.

It is important to note that some of the studies included in the review were of low quality. Additionally, a substantial portion of the reviews incorporated into our assessment relied on cross-sectional study designs. While cross-sectional studies offer valuable insights into associations at specific moments in time, they inherently lack the capacity to establish causation or capture changes over time. It is essential to address another limitation regarding the lack of discussion on mediating factors in the reviewed studies. While examining the risk factors associated with mental health disorders in patients with type 2 diabetes was the primary focus, the absence of in-depth exploration of mediating factors such as access to routine mental health support in clinic, therapeutic drugs, sedentary lifestyle, and unhealthy eating represents a significant gap in the analysis. Consequently, future research endeavours should prioritize the inclusion of comprehensive analyses focusing on potential mediating variables. Such an approach is essential for fostering a more nuanced comprehension of the multifaceted interplay among various factors influencing mental health outcomes.

Moreover, the lack of clarity in the scales employed for assessing depression in certain studies included in this review. This introduces a potential source of uncertainty, as the ambiguity in the scale used may result in challenges distinguishing between depression symptoms and a definitive diagnosis.

The small number of primary studies included in systematic reviews incorporated within this study may introduce constraints on the generalizability and depth of findings. As an example, only three primary studies examined BDNF levels, underscoring the limitations of drawing robust conclusions due to the limited amount of data available. This exemplifies the challenges associated with drawing robust conclusions, emphasizing the need for cautious interpretation due to the limited data available for analysis.

Finally, our search strategy was limited to studies specifically relevant to type 2 diabetes. This focused approach allowed us to focus on relevant research, but it carries the potential limitation of limited generalizability to other chronic diseases. Furthermore, our research was limited to the English language; therefore, we may have missed valuable research published in other languages.

## 8 Conclusion

In summary, this umbrella review identified twenty-six potential factors associated with mental health disorders, particularly depression in individuals with type 2 diabetes. Our comprehensive analysis demonstrated that obesity, neuropathy, complications, peripheral blood CRP concentrations, female sex, and social support are robust risk factors for mental health disorders in this population. These findings have significant clinical implications. For instance, increasing priority may be given to patients susceptible to or with these risk factors, categorizing them as high-risk groups for the development of mental health disorders. Establishing plans and policies accordingly may enhance support for these individuals before they develop these disorders. In addition, further research is needed to explore how various factors may mediate the association between type 2 diabetes and mental health disorders, such as blood concentrations of C-reactive protein (CRP). According to this study, the quality of evidence falls within Class III, which suggests it is relatively strong when compared to other diabetes indicators, such as Haemoglobin A1C.

## Funding

This research did not receive any specific grant from funding agencies on the public, commercial, or not-for-profit sectors.

## Availability of data

The datasets utilized in this study, which involved an umbrella review synthesizing data from existing systematic reviews and meta-analyses, have not been deposited into a publicly available repository. However, the datasets are available from the corresponding author upon reasonable request.

## Ethics approval and consent to participate

Not applicable.

## CRediT authorship contribution statement

**Amani Busili:** Writing – review & editing, Writing – original draft, Methodology, Formal analysis. **Kanta Kumar:** Writing – review & editing, Visualization, Validation, Supervision. **Laura Kudrna:** Writing – review & editing, Validation, Supervision. **Idris Busaily:** Writing – review & editing, Methodology, Data curation.

## Declaration of competing interest

The authors declare that they have no known competing financial interests or personal relationships that could have appeared to influence the work reported in this paper.
